# Quality of life and depression in caregivers of patients with breast cancer

**DOI:** 10.1186/1756-0500-5-310

**Published:** 2012-06-20

**Authors:** Mohammad Ali Heidari Gorji, Zinnatossadat Bouzar, Mohsen Haghshenas, Ali Akbar Kasaeeyan, Mohammad Reza Sadeghi, Maryam Didehdar Ardebil

**Affiliations:** 1 Department of nursing, Mazandaran University of Medical science, Sari, Iran; 2 Department of OB&GYN, Babol University of Medical Science, Babol, Iran; 3 Member of stem cell research center, Babol University of Medical Science, Babol, Iran; 4 Department of Pediatric, Babol University of Medical Science, Babol, Iran; 5 Department of Urology, Shahid Beheshti Hospital, Babol University of Medical Sciences, Babol, Iran; 6 Department of Psychology, Mazandaran University of Medical Sciences, Sari, Iran; 7 Clinical Psychology Department, Panjab University, Chandigarh, Iran

**Keywords:** Cancer, Care giver, Quality of life, Depression

## Abstract

**Background:**

Caregivers have a considerable role in caring and recovery of cancer patients. They may experience psychological problems such as depression, anxiety and decreases in quality of life (QOL). Present study aimed to explore depression and quality of life and their relationship among care givers of patients with breast cancer .

**Methods:**

In this cross sectional study, enrolled 63 care givers of women with breast cancer attending IMKH hospital in Iran as outpatients during 2009–2010. In order to assess the QOL and depression, we used Caregiver QOL Index-Cancer (CQOL-C) and Beck Depression Inventory respectively.

**Results:**

We found depression has strong negative correlation with QOL and participants with depression were more likely to have a poorer overall QOL.

**Conclusions:**

Depression has some effects on QOL of breast cancer patients’ care givers. Assistance and giving information through education and intervention from healthcare professionals is the key of improve the ability of caregivers to enhance their QOL.

## Background

Cancer patients’ caregivers may be affected by various stressors such as psychological, social, or physical health functioning. Behaviors such as diminished rest or exercise and neglecting their own due to care from a patient who has breast cancer, can influence their health and quality of life [1,2]. On the other hand caregiver’s mentality and quality of life are significantly affected by a patient’s stage of illness [3,4]. Previous literatures showed depression is greater in cancer caregivers than in the general population [5,6], and caring from cancer patients can increase a risk for depression, anxiety, sleep disruption and finally diminish QOL [7-11]. A meta-analysis of psychological distress among cancer patients and family caregivers found that both members of the dyad experienced similar levels of distress [12]. While conversely in some studies reported family members of cancer patients do not have clinically problematic emotional distress [13]. But sometime caregivers experience a complex powerful emotion that may be equal or surpass those experienced by the patient during diagnosis and treatment process [14,15], although caregiver’s problems are considered in some studies but overall less attention has been paid to cancer caregivers’. There is some contrast in previous studies and furthermore, as researchers knowledge, no studies have simultaneously evaluated the prevalence and correlates of depression or their relevance to quality of life in breast cancer patient’s caregivers in Iran. Accordingly, the specific aims of this study were to examine the correlates of depression in relation to quality of life among breast cancer caregivers.

## Methods

This study has used a cross-sectional descriptive design. The sample was selected by convenience method from Clinical Oncology Departments of Imam Khomeini hospital in Iran. A total of 63 caregivers were sought for current study. Inclusion Criteria: caregivers had to be providing care for adult patients with breast cancer, and willing to participate in this study. All participants agreed to participate and signed a consent form approved by the Research Ethics Committee of the University Faculty of Medicine.

### Tools

The considered variables in demographic questionnaire were age, income, marital status, educational level, and employment status, family history of cancer and stage of disease.

The Caregiver Quality of Life Index-Cancer (CQOL-C) was used to assess the quality of life, which is 35 items scored by using a five-point Likert-type scale that yields a single QOL score. Items assess the impact of cancer on the caregiver's mood, worry, sleep, daily life, family life, and other dimensions. The instrument is psychometrically sound and has been used with hospice care giving samples [16].

To assess depression used the Beck Depression Inventory (BDI), which was originally designed to measure depression in mentally ill patients and evaluates 21 symptoms of depression. Each question is rated on a four-point intensity scale and total scores range from 0 to 63. Higher scores mean more severe depression. We used the following cutoff scores for the BDI: no or minimal depression, <10; mild-to-moderate depression, 10–18; moderate-to-severe depression, 19–29; and severe depression, 30–63 [17]. In this study care givers defined as one of the family members who give most assistance in patient’s activities of daily living.

### Analysis

Descriptive statistics run for all data to obtain means, standard deviations, frequencies and percentages. The correlation coefficient was used to explore whether the QOL showed difference with different level of depression or not. We used the Statistical Package for Social Science (SPSS version 14.0) to analyze the data. A p level <05 was considered to be significant.

## Results and discussion

Descriptive analysis of data indicates that out of the total sample mild to severe depressive symptoms were found in 60% (24.8% mild and rest moderate and sever) of patients’ care givers. There was 45% smoking and no drug abusing in our sample. Among 63 selected subjects, 26 cases (41.3) were male and 37 (58.7) were female. Mean age was 52/48 _ + 14/04. 73% of patients were working and 11% were retired and rest of patients was non-working. All participants were literate, 50.6 had elementary education level and rest higher. 48% of care givers had chronic disease mostly diabetes (28%). Among participants 17.5% had life quality lower than normal and 42.9% were medico rite, only 39.7% of patients were higher than appropriate (Table 1). Negative correlation was strong between caregiver depression and quality of life (*r* = − 0.67;*p* < 0.01), and found correlation between depression and income (*r* = 0.53;*p* < 0.01), education(*r* = 0.36;*p* < 0.05) also(Table 2).

**Table 1:** Frequency of QOL and Depression

**Variables/Frequency &Percentage**	**QOL**	**Depression**
Weak	11 (17.5%)	
Moderate	27 (42.9%)	
Good	25 (39.7%)	
No or minimal depression <10		25(39.7%)
Mild-to-moderate (10–18)		15(24.8%)
Moderate-to-severe depression (19–29)		15(24.8%)
Severe depression(30–63)		8(10.7%)

**Table 2:** Correlation of QOL and Depression

**QOL**	**R**	**P**
QOL (totally)	./67	<0/01
Education	./36	<0/05
Income	./53	<0/01
Age	./33	<0/22

## Discussion

Authors explored the prevalence and correlates of depression and QOL in care givers of patients with breast cancer. Some of the cancer patients’ care givers were disappointed and hopeless. Mental condition changes of patient’s can influences family members too, and results to inactivation of any productive actions. Findings were demonstrated that high percent of caregivers were afflicted by mild and moderate depression. Cameron and Beach also reported same results [18,19]. Depression, even in mild level, disturbs the mental health. In this study depression was somewhat higher than other studies. The reason may be because our subjects (48%) of care givers suffered from their own chronic disease also. In term of life quality, results showed that 42% and 11% reported moderate and low quality of life, respectively. Health and Hellstrom also reported that improper life quality in care givers of cancer patients [20,21]. And there were correlation between depression, quality of life, income and education. In fact the problem such as depression is a substantial and common among cancer patients. These problems can affect the personal relations, clinical course and prognosis of patients’ disease [22] and quality of life in whole of family. Finally, concerning effect of depression on quality of life and inversely by improving the life quality of patients, besides prevention chance of recovery in patients can be increased, too.

## Conclusion

The results of this study demonstrate that psychological issues have a significant impact on quality of life. Additional help and attention to caregivers would be beneficial in improving quality of life of all family of patients. Lack of special attention to caregivers is a serious gap in health care.

It is essential that descriptive and longitudinal designs be consider the care requirements. Further studies should take into consideration on safety, risk for negative outcomes, and adverse effects for both the caregiver and patients be noted. Finally, interventions must be designed and introduced to professional or formal caregivers and family caregivers who offer vital skills and resources.

## Abbreviations

CQOL-C, Quality of Life Index-Cancer; BDI, Beck Depression Inventory.

## Misc

Mohammad Ali Heidari Gorji, Mohsen Haghshenas, Ali Akbar Kasaeeyan and Maryam Didehdar Ardebil contributed equally to this work.

## Competing interests

The authors declare that they have no competing interests.

## Authors’ contributions

MHG and MS contributed to the study design and drafting. Data acquisition was carried out by MH contributed to data analysis. ZB helped in data collection. MD and AK revised the manuscript. All authors read and approved the final version of the manuscript.

## Authors’ information

MHG is assistant professor and AK and MS are member faculty of Mazandaran University, Iran. ZB is associated professor and MH is assistant professor of Babol Medical science University, Iran. MD is PhD scholar student in Punjab University of India.
